# Genetic diversity and epidemiology of human rhinovirus among children with severe acute respiratory tract infection in Guangzhou, China

**DOI:** 10.1186/s12985-021-01645-6

**Published:** 2021-08-23

**Authors:** Wanwei Li, Bo Yu, Jijian Zhou, Yanlan Wang, Bao Xue, Jialong Pan, Yanhong Ran, Xiaoping Yang, Xiaoping Wang, Fang Yang, Hongjian Li

**Affiliations:** 1 The Key Laboratory for Virology of Guangzhou, Department of Biotechnology, College of Life Science and Technology, Jinan University, Guangzhou, Guangdong China; 2 First Affiliated Hospital of Jinan University, Guangzhou, Guangdong China; 3 Institute of Medical Microbiology, Jinan University, Guangzhou, China; 4 The Key Laboratory for Virology of Guangdong, Jinan University, Guangzhou, China

**Keywords:** Human rhinovirus, Genetic diversity, Symptom severity, Viral load

## Abstract

**Background:**

Human rhinovirus (HRV) is one of the major viruses of acute respiratory tract disease among infants and young children. This work aimed to understand the epidemiological and phylogenetic features of HRV in Guangzhou, China. In addition, the clinical characteristics of hospitalized children infected with different subtype of HRV was investigated.

**Methods:**

Hospitalized children aged < 14 years old with acute respiratory tract infections were enrolled from August 2018 to December 2019. HRV was screened for by a real-time reverse-transcription PCR targeting the viral 5′UTR.

**Results:**

HRV was detected in 6.41% of the 655 specimens. HRV infection was frequently observed in children under 2 years old (57.13%). HRV-A and HRV-C were detected in 18 (45%) and 22 (55%) specimens. All 40 HRV strains detected were classified into 29 genotypes. The molecular evolutionary rate of HRV-C was estimated to be 3.34 × 10^–3^ substitutions/site/year and was faster than HRV-A (7.79 × 10^–4^ substitutions/site/year). Children who experienced rhinorrhoea were more common in the HRV-C infection patients than HRV-A. The viral load was higher in HRV-C detection group than HRV-A detection group (*p* = 0.0148). The median peak symptom score was higher in patients with HRV-C infection as compared to HRV-A (*p* = 0.0543), even though the difference did not significance.

**Conclusion:**

This study revealed the molecular epidemiological characteristics of HRV in patients with respiratory infections in southern China. Children infected with HRV-C caused more severe disease characteristics than HRV-A, which might be connected with higher viral load in patients infected with HRV-C. These findings will provide valuable information for the pathogenic mechanism and treatment of HRV infection.

## Background

Human rhinoviruses (HRV) are the most commonly encountered respiratory viruses and the most frequent causes of acute respiratory infections in young children and infants. HRV is generally associated with common cold and mild upper respiratory infections, but may also lead to more severe lower respiratory tract illnesses, such as pneumonia, bronchiolitis and asthma [1, 2].

HRV belongs to the genus Enterovirus and family Picornaviridae, and currently has more than 100 serotypes, classified into three species: HRV-A, B and C. Species HRV-A and HRV-B were discovered in the 1950s [3], but HRV-C was identified in 2006 using novel molecular-based techniques [4, 5]. There are more than 100 distinct serotypes of HRVs identified according to their capsid proteins. All HRV species have been identified in throughout the year in many regions, and HRV-A and HRV-C appear to be the predominant species detected in patients with acute respiratory infection [6]. Recent studies suggest that the illness severity differs among HRV species [7, 8], and HRV-C has been shown more frequently associated with severe asthma attacks and lower respiratory tract infections compared with other HRV species [9–11]. Also, recently studies have shown that HRV-C is possibly more virulent and cause more severe illness [8, 12–14].

In addition, the association of HRV species with clinical severity is not well understood. In this study, we investigated the epidemiological, evolution, and clinical characteristics of HRV infections in children with acute respiratory tract infections. Furthermore, we studied the impact of HRV species and nasopharyngeal viral load on the disease severity of acute respiratory tract infections.

## Methods

### Study population and sample collection

A total of 655 nasopharyngeal swab specimens were collected from hospitalized infants or children with acute respiratory tract infection from Guangdong Panyu Maternal and Child Health Hospital between August 2018 and December 2019. Specimens were only taken from individuals primary diagnosis of viral infection and with $\leqslant$ 3 days of fever (temperature $\geqslant$ 37.5 °C), cough, sputum, throat sore, dyspnea and/or other acute respiratory tract infection symptoms. Nasopharyngeal swabs were collected within 24 h after admission by medical professionals, and all the samples were placed in viral transportation medium and stored at − 80 °C. Demographic information and clinical characteristics were recorded for each patient. The degree of disease severity of each HRV infection patient was estimated and scored according to a severity scoring system as described previously [15].

### Detection of respiratory viruses

Total viral nucleic acids were extracted from the viral transportation medium using the QIAamp MinElute Virus Spin Kit (Qiagen, Valencia, CA) according to the manufacturer’s instructions. Reverse transcription was performed using RevertAid First Strand cDNA Synthesis kit (Invitrogen Life Technologies, Carlsbad, CA) with random primers. The cDNA was used for virus detection immediately or stored at -20˚C until further use. HRV infection was detected by using qRT-PCR with HRV specific primers and a probe, HRV forward primer (5′-TGGACAGGGTGTGAAGAGC-3′), reverse primer (5′-CAAAGTAGTCGGTCCCATCC-3′), and probe (FAM-TCCTCCGGCCCCTGAATG-BHQ1). Other viral infections were simultaneously screened, including human parainfluenza virus, influenza virus, respiratory syncytial virus, human coronaviruses (229E, NL63, HKU1, and OC43), metapneumovirus, adenovirus, and bocavirus.

### Gene sequencing

Samples that tested positive for HRV were used to further determine genotypes, the VP4/VP2 and 5′UTR regions of HRV were amplified using nest-PCR, primers were synthesized from the published primer sequences [16]. PCR was initiated at 95 °C for 10 s, followed by 35 cycles of 95 °C for 5 s, 55 °C for 30 s and 72 °C for 1 min, with a final extension at 72 °C for 10 min. Specimens from which amplification of the VP4/VP2 regions failed were defined as untyped. All sequencing was performed by Sangon Biotech Co., Ltd. (Shanghai, China) using ABI-PRISM 3730XL DNA sequencer (Applied Biosystems).

### Phylogenetic analyses by neighbour-joining (NJ) and Bayesian Markov Chain Monte Carlo (MCMC) methods

The sequences obtained in this study were aligned with representative sequences retrieved from GenBank using Clustal W. The phylogenetic tree was constructed using the NJ method in MEGA 7.0 software [17], and the reliability of the tree was estimated with 1000 bootstrap replications.

Molecular evolutionary analysis was performed with Bayesian Markov Chain Monte Carlo (MCMC) method using BEAST ver.2.5.1 [18]. The most suitable nucleotide substitution model (GTR + G) was selected using jModelTest 2.1.10 [19], and the datasets were analyzed with an uncorrelated lognormal relaxed clock model. Convergence was assessed using Tracer version 1.7.1, and it was accepted when the MCMC chain was run through enough steps to make the effective sample size (ESS) above 200 after a 10% burn-in. The maximum clade credibility (MCC) tree was constructed by Tree Annotate 2.5.1 after removing the first 10% of trees as burn-in, and the phylogenetic tree was visualized by FigTree v1.4.4. The uncertainties of the estimates were indicated by 95% highest posterior density (HPD) intervals.

### Statistical analysis

Continuous variables were analyzed with the one-way analysis of variance (ANOVA) and Student’s t-test; chi-square was performed for ordinal or categorical data. Mann–Whitney U-test was used to compare severity scores between groups. A *p* value below 0.05 was considered statistically significant. Statistical analysis was performed with SPSS software (version 17.0; SPSS, Inc., Chicago, IL, USA).

## Results

### Demographic characteristics and seasonal distribution of HRV infection

**Fig. 1:**
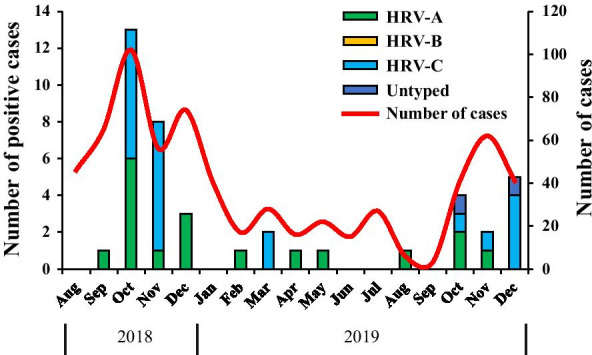
Seasonal distribution of HRV species in Guangzhou, China, between August 2018 and December 2019

A total of 655 nasopharyngeal swab specimens were collected from children with acute respiratory illness. Of these, real-time PCR revealed that 42 (6.41%) of 655 hospitalized children were HRV positive, and there is no HRV-positive patients were co-infected with other respiratory viruses. As shown in Table 1, approximately 90.5% (38/42) of these children were under 5 years of age, and the majority of them were under 2 years old (57.13%). The age and gender distributions of the HRV-positive patients were not statistically significant. The prevalence of HRV infections during August 2018 to December 2019 in Guangzhou is shown in Fig. 1. Among the collected specimens, HRV-A was identified in 42.9% of samples (18/42), HRV-C in 52.4% (22/42) and HRV-B was not identified. In addition, there are two samples that were not successfully typed. HRV was prevalent mainly in the winter, especially in October to November.

**Table 1:** Demographic of children hospitalized with HRV infection

Characteristics	Number of patient	Number of HRV infected children	*p* value
	N = 655	N = 42	
	n (%)	n (%)	
*Age groups*			
≤ 1	189 (28.85)	15 (35.71)	0.365^a^
1–2	155 (23.66)	9 (21.42)	0.735^a^
2–3	92 (14.04)	6 (14.28)	1^a^
3–4	89 (13.58)	5 (11.90)	1^a^
4–5	46 (7.02)	3 (7.14)	1^a^
> 5	84 (12.82)	4 (9.52)	0.658^a^
*Gender*			
Male	379 (57.8)	20 (47.6)	0.202^a^
Female	276 (42.2)	22 (52.4)	

*HRV* human rhinovirus

^a^Conducted by chi-squared test

### Phylogenetic analysis of HRV strains using the neighbor joining method

**Fig. 2:**
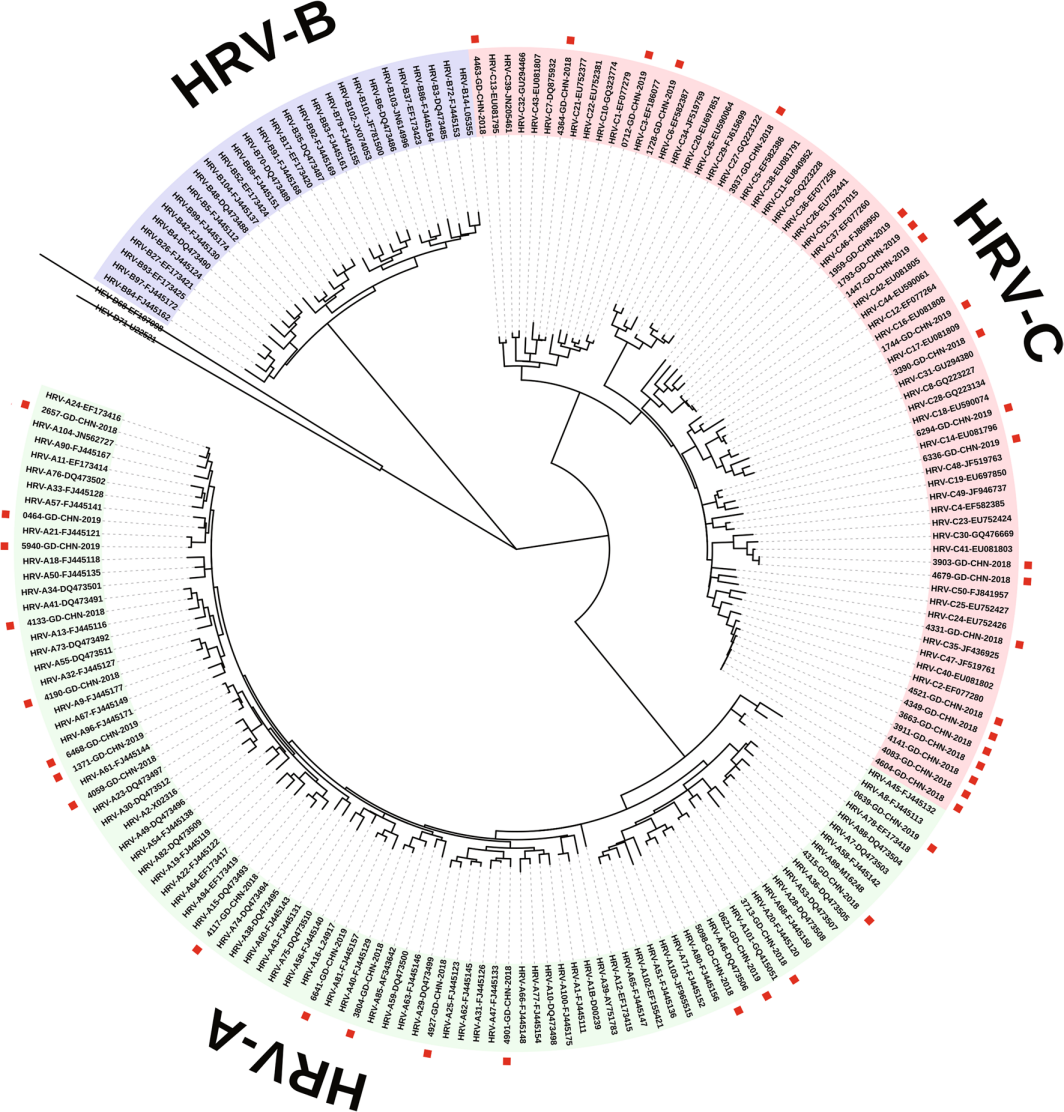
Phylogenetic tree for VP4/VP2 region of HRV. The tree was constructed using Maximum Likelihood method with MEGA 7.0 based on 1000 bootstrap replicates. Reference strains representing known genotypes were retrieved from GenBank. The Guangzhou HRV isolates are indicated by “A specific number-GD-CHN-year”. The sequences detected in the present study are followed by a red square

The VP4/VP2 region (approximately 420 bases) was sequenced for 40 real-time PCR positive samples. In order to confirm the classification of HRV types of the 40 sequences, phylogenetic analysis of the VP4/VP2 region sequences was performed. As shown in Fig. 2, the HRV-A strains for each genotype were as follows: A15 (1); A16 (1); A21 (1); A24 (1); A29 (1); A36 (1); A40 (1); A41 (1); A46 (1); A47 (1); A57 (1); A61 (3); A78 (1); A80 (1); A101 (1). The present HRV-C strains in each genotype were as follows: HRV-C2 (7); HRV-C3 (1); HRV-C5 (1); HRV-C6 (1); HRV-C13 (1); HRV-C14 (1); HRV-C17 (1); HRV-C20 (1); HRV-C21 (1); HRV-C31 (1); HRV-C35 (1); HRV-C41 (2); HRV-C46 (3); HRV-C48 (1).

**Fig. 3:**
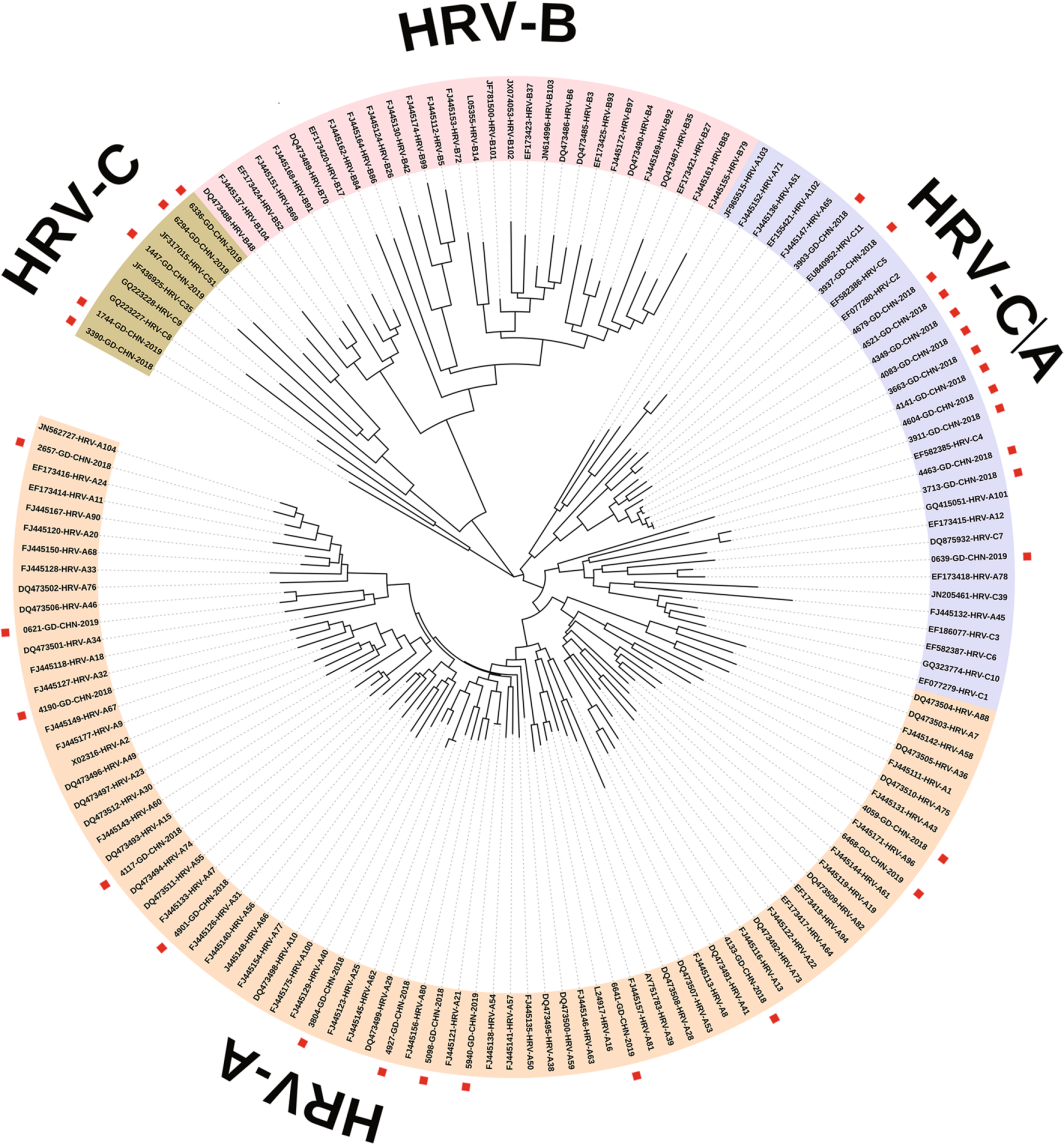
Phylogenetic tree for 5’-UTR region of HRV. The tree was constructed using Maximum Likelihood method with MEGA 7.0 based on 1000 bootstrap replicates. Reference strains representing known genotypes were retrieved from GenBank. The Guangzhou HRV isolates are indicated by “A specific number-GD-CHN-year”. The sequences detected in the present study are followed by a red square

The phylogenetic analysis of the 5’UTR sequences was also performed. As shown in Fig. 3, HRV-B and majority of HRV-A sequences formed a distinct clade, while the tree formed a clade that includes intermixed HRV-A and HRV-C strains. All HRV-positive samples were grouped into 18 genotypes, and one sample (3903-GD-CHN-2018) was not typed. Therefore, our observations indicate that the main species of HRV circulating in Guangzhou from 2018 to 2019 were HRV-A and HRV-C, whereas the prevalent genotype was HRV-C2. Moreover, multiple HRV serotypes could be detected within a time period.

### Estimation of the time to the most recent common ancestor (tMRCA) and molecular evolutionary rate for HRV strains using the Bayesian MCMC method

**Fig. 4:**
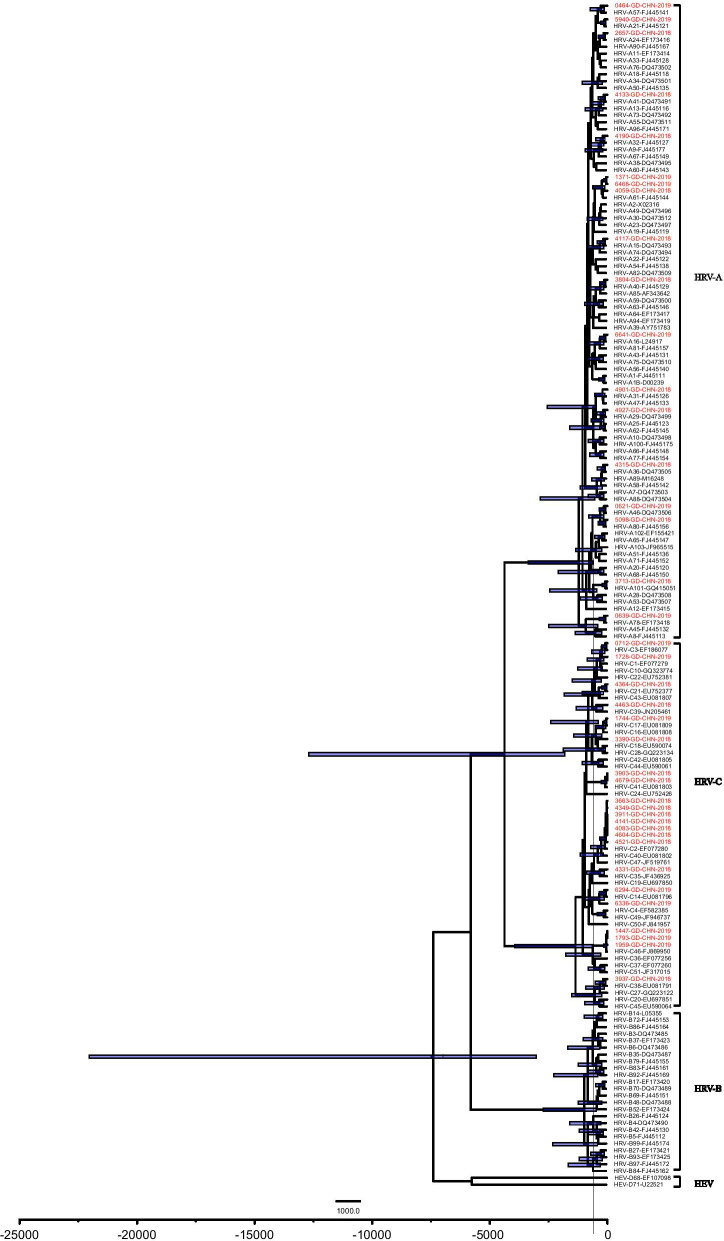
Time-scaled Bayesian maximum clade credibility (MCC) tree of the VP4/VP2 coding region for HRV with Bayesian Markov Chain Monte Carlo (MCMC). HRV strains from the present study are colored red. Blue bars indicate 95% highest posterior density (HPD) for the estimated year. Only posterior probabilities of > 0.90 are shown at the branch nodes

The phylogenetic trees with Bayesian Markov Chain Monte Carlo (MCMC) method as shown in Fig. 4 were constructed to estimate the time-scaled evolution of HRV VP4/VP2 region for HRV global strains. The time-scaled Maximum Clade Credibility (MCC) tree revealed that the times to the most recent common ancestor (tMRCAs) of HRV-A was around 1310 years ago (95% highest probability density (HPD): 590–3396) and 1370 years ago (95% HPD: 590–3962) for HRV-C.

The molecular evolutionary rate of HRV-A strains was estimated to be 7.79 × 10^–4^ substitutions/site/year (95% HPD: 2.87 × 10^–5^-1.89 × 10^–4^), while that of HRV-C was estimated to be 3.34 × 10^–3^ substitutions/site/year (95% HPD: 1.63 × 10^–3^ to 5.26 × 10^–3^). The rate of HRV-C strains was significantly faster than that of HRV-A strains.

### Clinical and laboratory characteristics in children with HRV infection

To characterize the clinical manifestations caused by each HRV species, the clinical symptomsand laboratory tests of HRV-positive patients are listed in Table 2. All HRV-positive patients presented with severe acute respiratory infection (SARI). Cough (87.5%), rhinorrhoea (62.5%), fever (62.5%) and expectoration (55%) were the most common symptoms at presentation. In addition, HRV-C positive patients more often were expectoration (*p* = 0.026) as compared to HRV-A positive patients. In other clinical features, no statistically significant difference was recorded.

**Table 2:** Clinical and laboratory characteristics among HRV-positive hospitalized children

Characteristics	HRV-A (n = 18)	HRV-C (n = 22)	P-value
Age (months) -median (IQR)	21.5 (11.25–37.75)	16 (9–23.5)	0.184^a^
Male sex-no. (n, %)	8 (44.4%)	12 (57%)	0.751^b^
Clinical symptoms and signs			
Fever (n, %)	14 (77.8%)	11 (50.0%)	0.104^b^
Cough (n, %)	15 (83.3%)	20 (90.9%)	0.642^b^
Nasal obstruction (n, %)	2 (11.1%)	7 (31.8%)	0.149^b^
Wheezing (n, %)	6 (33.3%)	8 (36.4%)	1^b^
Rhinorrhoea (n, %)	12 (66.7%)	13 (59.1%)	0.747^b^
Expectoration (n, %)	6 (33.3%)	16 (72.7%)	0.024^b^
Sneeze (n, %)	3 (16.7%)	2 (9.1%)	0.649^b^
Heart rate/min-median (IQR)	112 (108.8–123.5)	116 (107–125)	0.916^a^
Respiratory rate/min-median (IQR)	26.5 (23.5–30)	28 (25.5–32)	0.206^a^
Laboratory tests			
Leukocyte (× 10^9^/L)-median (IQR)	11.31(7.48–15.16)	10.86 (7.26–13.84)	0.52^a^
Neutrophil percentage (%)-median (IQR)	40.4 (25.65–65.3)	36.3 (27.25–62.65)	0.928^a^
Lymphocyte percentage (%)-median (IQR)	47.7 (29.08–64.1)	46.5 (27.9–62.55)	0.815^a^
Clinical outcomes and treatment			
Bronchopneumonia (%)	12 (66.7%)	14 (63.6%)	1^b^
Antibiotic use (%)	5 (27.8%)	6 (27.6%)	1^b^

^a^Conducted by nonparametric Mann–Whitney U text

^b^Conducted by chi-squared test

### Viral load and severity of infection

**Fig. 5:**
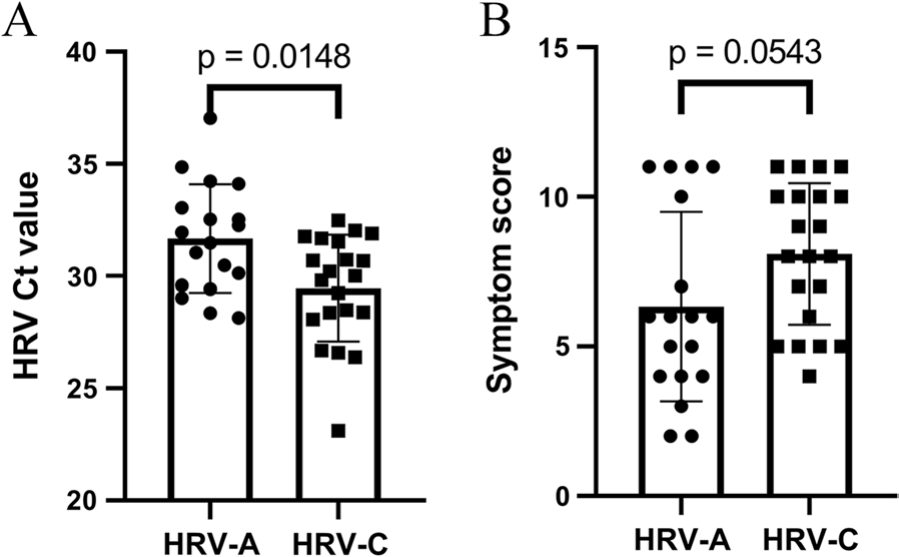
Scatter plots of HRV real-time reverse-transcription polymerase chain reaction cycle threshold (Ct) values and symptom scores of patients with HRV-A and HRV-C infections. The comparison of viral load and symptom score were conducted by nonparametric Mann–Whitney U-test

To determine the relationship between the viral load of different HRV genotypes and the severity of the disease. We compared median symptom score and Ct values for each HRV species. As shown in Fig. 5, the Ct value of HRV-A ranged from 28.15 to 37.05 and HRV-C ranged from 23.1 to 32.47. The viral load of HRV-A was significantly higher than that of HRV-C (*p* = 0.0148). According to the severity scoring system, the median peak symptom score for the 18 patients with HRV-A infection was 6 (range 4–10.25), and that 22 patients with HRV-C infection was 8 (range 5.5–10). And the median peak symptom score was higher in patients with HRV-C infection as compared to HRV-A (*p* = 0.0543), although the difference did not reach significance.

## Discussion

HRV is the predominant pathogen identified in hospitalized infants and young children with acute respiratory tract infection [1]. HRV although mostly associated with mild disease, can also be a cause of severe illness [20]. In this study, we analyzed the epidemiology and genotypic diversity of HRV within hospitalized children. During the monitoring, 42 (6.41%) were positive for HRV, which is consistent with previous research conducted in Guangdong (5.47%) [21]. However, the prevalence of HRV could be different in across regions and years [22–24].

The highest incidence of HRV infection occurred among the 0–1-year-old infants, and 71.4% (30/42) of total HRV occurred in children younger than 3 years old. This is comparable with the results found in China and other countries [13, 24–26]. These observations support the view that infants were more susceptible to HRV.

The phylogenetic analysis shows that only HRV-A and HRV-C are circulating in children in the population we analyzed. The proportion of the HRV species revealed in this study (HRV-A, 45% and HRV-C, 55%) is consistent with prior studies [2, 23, 27, 28]. The evolutionary rate of viral genes differs among respiratory viruses as previous reported [29, 30]. In the present study, the evolutionary rate of the VP4/VP2 region of HRV-A (3.34 × 10^–3^ substitutions/site/year) was faster than that reported for global HRV-C strains (6.6 × 10^–4^ substitutions/site/year) [31], but similar to that reported for Japan HRV-C strain(3.07 × 10^–3^ substitutions/site/year) [32]. Therefore, the evolution rate of HRV strains varies from region to region, but the precise mechanisms are not known yet.

Clinical symptoms of HRV infected patients were similar to those previously reported cough, rhinorrhoea, fever and expectoration [33]. In this study there were no significant differences observed in clinical symptoms between two species, except more frequent expectoration in patients with HRV-C species. Previous studies have reported that HRV-C causes more severe clinical manifestations compared with the other HRV types [8, 12, 34]. And some observations suggested that in HRV-C infected patients with pneumonia, higher mean viral load was reported [35]. However, another research shown there was no significant difference in median peak viral load between HRV species in hospitalized patients [36]. In the present analysis, we observed that HRV-C viral load in hospitalized children was significantly higher than HRV-A, indicate that association between HRV species and viral load was found. Interestingly, HRV-C infected patient exhibits higher symptom score in comparison to patient infected with HRV-A. Although statistically significant differences was not found, the disease was more severe in patients infected with HRV-C comparison with HRV-A. Together, our findings suggest that the pathophysiology of infection with HRV-C may differ from HRV-A. Previous studies have demonstrated that HRV-A and HRV-B utilize the intercellular adhesion molecule 1 (ICAM-1) and low-density lipoprotein receptor (LDLR) family members as cellular receptor [37, 38]. A unique feature of HRV-C is its use of the cadherin-related family member 3 (CDHR3) for host cell entry. Moreover, a single-nucleotide polymorphism (C529) in CDHR3 is associated with enhanced viral binding and promoting viral replication with a consequent increase in viral load [39]. Further study will be needed to determine the mechanism of HRV-A and HRV-C resulting in different disease severities.

## Conclusion

In conclusion, we analyzed the molecular characterization of HRV and indicated that HRV-A and HRV-C were predominant in HRV infections. The genetic variability analysis of HRV highlights the genetic complexity and rapid evolution of HRV that warrant continuous molecular surveillance. Clinical data analysis reveals HRV-C and high viral load were shown to be the important determinants of the severity of acute respiratory illnesses. Our study highlight the fact that it is necessary to study the mechanism of specific HRV genotype in causing severe disease.

## Data Availability

The datasets used and/or analyzed in the current study are available from the corresponding author upon reasonable request.

## Abbreviations

HRV: human rhinovirus
SARIs: severe acute respiratory infections
qRT-PCR: real-time reverse transcription polymerase chain reaction
MEGA: molecular evolutionary genetics analysis
NJ: neighbour-joining method
SPSS: statistical product and service solutions
ICAM-1: intercellular adhesion molecule 1
LDLR: low-density lipoprotein receptor
CDHR3: cadherin-related family member 3
MCMC: Markov Chain Monte Carlo
ESS: effective sample size
MCC: maximum clade credibility
HPD: highest posterior density
ANOVA: one-way analysis of variance
tMRCAs: times to the most recent common ancestor

## References

[1] Nair H (2013). Global and regional burden of hospital admissions for severe acute lower respiratory infections in young children in 2010: a systematic analysis. The Lancet.

[2] Rahamat-Langendoen JC (2013). The significance of rhinovirus detection in hospitalized children: clinical, epidemiological and virological features. Clin Microbiol Infect Off Publ Eur Soc Clin Microbiol Infect Dis.

[3] Price WH (1956). The isolation of a new virus associated with respiratory clinical disease in humans. Proc Natl Acad Sci U S A.

[4] Lamson D, Renwick N, Kapoor V, Liu Z, Palacios G, Ju J, Dean A, St George K, Briese T, Lipkin WI (2006). MassTag polymerase-chain-reaction detection of respiratory pathogens, including a new rhinovirus genotype, that caused influenza-like illness in New York State during 2004–2005. J Infect Dis.

[5] Lau SK (2007). Clinical features and complete genome characterization of a distinct human rhinovirus (HRV) genetic cluster, probably representing a previously undetected HRV species, HRV-C, associated with acute respiratory illness in children. J Clin Microbiol.

[6] Arakawa M (2012). Molecular epidemiological study of human rhinovirus species A, B and C from patients with acute respiratory illnesses in Japan. J Med Microbiol.

[7] Calvo C (2009). Role of rhinovirus C in apparently life-threatening events in infants, Spain. Emerg Infect Dis.

[8] Khetsuriani N (2008). Novel human rhinoviruses and exacerbation of asthma in children. Emerg Infect Dis.

[9] Linsuwanon P (2009). High prevalence of human rhinovirus C infection in Thai children with acute lower respiratory tract disease. J Infect.

[10] Bizzintino J (2011). Association between human rhinovirus C and severity of acute asthma in children. Eur Respir J.

[11] Gern JE (2010). The ABCs of rhinoviruses, wheezing, and asthma. J Virol.

[12] Cox DW (2013). Human rhinovirus species C infection in young children with acute wheeze is associated with increased acute respiratory hospital admissions. Am J Respir Crit Care Med.

[13] Iwane MK (2011). Human rhinovirus species associated with hospitalizations for acute respiratory illness in young US children. J Infect Dis.

[14] Lee WM (2012). Human rhinovirus species and season of infection determine illness severity. Am J Respir Crit Care Med.

[15] Gern JE (2002). Relationships among specific viral pathogens, virus-induced interleukin-8, and respiratory symptoms in infancy. Pediatr Allergy Immunol.

[16] Wisdom A (2009). Screening respiratory samples for detection of human rhinoviruses (HRVs) and enteroviruses: comprehensive VP4–VP2 typing reveals high incidence and genetic diversity of HRV species C. J Clin Microbiol.

[17] Kumar S (2016). MEGA7: molecular evolutionary genetics analysis version 7.0 for bigger datasets. Mol Biol Evol.

[18] Bouckaert R (2014). BEAST 2: a software platform for Bayesian evolutionary analysis. PLoS Comput Biol.

[19] Darriba D (2012). jModelTest 2: more models, new heuristics and parallel computing. Nat Methods.

[20] Drysdale SB (2017). Rhinovirus: not just the common cold. J Infect.

[21] Luo HJ (2020). Epidemiological characteristics and phylogenic analysis of human respiratory syncytial virus in patients with respiratory infections during 2011–2016 in southern China. Int J Infect Dis Off Publ Int Soc Infect Dis.

[22] Zheng SY (2018). Epidemiological analysis and follow-up of human rhinovirus infection in children with asthma exacerbation. J Med Virol.

[23] Zhao Y (2018). Genotypic diversity and epidemiology of human rhinovirus among children with severe acute respiratory tract infection in Shanghai, 2013–2015. Front Microbiol.

[24] Yan Y (2017). Clinical and epidemiological profiles including meteorological factors of low respiratory tract infection due to human rhinovirus in hospitalized children. Ital J Pediatr.

[25] Zeng SZ (2014). Prevalence of human rhinovirus in children admitted to hospital with acute lower respiratory tract infections in Changsha, China. J Med Virol.

[26] Linder JE (2013). Human rhinovirus C: age, season, and lower respiratory illness over the past 3 decades. J Allergy Clin Immunol.

[27] Lauinger IL (2013). Patient characteristics and severity of human rhinovirus infections in children. J Clin Virol Off Publ Pan Am Soc Clin Virol.

[28] Jacobs SE (2015). Clinical and molecular epidemiology of human rhinovirus infections in patients with hematologic malignancy. J Clin Virol Off Publ Pan Am Soc Clin Virol.

[29] Mizuta K (2011). Detailed genetic analysis of hemagglutinin-neuraminidase glycoprotein gene in human parainfluenza virus type 1 isolates from patients with acute respiratory infection between 2002 and 2009 in Yamagata Prefecture, Japan. Virol J.

[30] Peck KM, Lauring AS (2018). Complexities of viral mutation rates. J Virol.

[31] Briese T (2008). Global distribution of novel rhinovirus genotype. Emerg Infect Dis.

[32] Kiyota N (2014). Genetic analysis of human rhinovirus species A to C detected in patients with acute respiratory infection in Kumamoto Prefecture, Japan 2011–2012. Infect Genet Evol J Mol Epidemiol Evol Genet Infect Dis.

[33] Peltola V (2009). Rhinovirus infections in children: a retrospective and prospective hospital-based study. J Med Virol.

[34] Pierangeli A (2013). Molecular epidemiology and genetic diversity of human rhinovirus affecting hospitalized children in Rome. Med Microbiol Immunol.

[35] Choi SH (2015). Clinical and molecular characterization of rhinoviruses A, B, and C in adult patients with pneumonia. J Clin Virol Off Publ Pan Am Soc Clin Virol.

[36] Piralla A (2009). Clinical severity and molecular typing of human rhinovirus C strains during a fall outbreak affecting hospitalized patients. J Clin Virol Off Publ Pan Am Soc Clin Virol.

[37] Greve JM (1989). The major human rhinovirus receptor is ICAM-1. Cell.

[38] Hofer F (1994). Members of the low density lipoprotein receptor family mediate cell entry of a minor-group common cold virus. Proc Natl Acad Sci U S A.

[39] Bochkov YA (2015). Cadherin-related family member 3, a childhood asthma susceptibility gene product, mediates rhinovirus C binding and replication. Proc Natl Acad Sci U S A.

